# A study comparing the experiences of family and friends of young people with borderline personality disorder features with family and friends of young people with other serious illnesses and general population adults

**DOI:** 10.1186/s40479-020-00128-4

**Published:** 2020-07-22

**Authors:** Mirra R. Seigerman, Jennifer K. Betts, Carol Hulbert, Ben McKechnie, Victoria K. Rayner, Martina Jovev, Sue M. Cotton, Louise McCutcheon, Catharine McNab, Emma Burke, Andrew M. Chanen

**Affiliations:** 1 Melbourne School of Psychological Sciences, The University of Melbourne, Melbourne, Australia; 2 Orygen, Melbourne, Australia; 3 Centre for Youth Mental Health, The University of Melbourne, Melbourne, Australia

**Keywords:** Carers, Borderline personality disorder, Psychological distress, Experiences of caregiving, Coping, Expressed emotion, Youth, Adolescent

## Abstract

**Background:**

Family and friends (‘carers’) of adults with borderline personality disorder (BPD) and carers of young people with other serious illnesses experience significant adversity but research on the experiences of caring for a young person with BPD features is sparse. This study aimed to: (i) describe the experiences of carers of young people with BPD features; (ii) compare them with published data assessing carers of young people with other serious illnesses and with adults from the general population.

**Methods:**

Eighty-two carers (M age = 44.74, SD = 12.86) of 54 outpatient young people (M age = 18.76, SD = 3.02) who met 3 to 9 DSM-IV BPD criteria completed self-report measures on distress, experiences of caregiving, coping, and expressed emotion. Independent-samples t-tests were employed to compare scores with those reported by convenience comparison groups of general population adults or carers of young people with eating disorders, cancer, or psychosis.

**Results:**

Carers of young people with BPD features reported significantly elevated levels of distress, negative caregiving experiences, and expressed emotion, as well as maladaptive coping strategies, compared with general population adults or carers of young people with other serious illnesses.

**Conclusions:**

Carers of young people with BPD features experience elevated levels of adversity compared with their peers in the general adult population. This adversity is similar to, or greater than, that reported by carers of young people with other severe illnesses. Research is needed to clarify factors underlying adverse caregiving experiences and to develop and evaluate interventions to support carers of young people with BPD features.

**Trial registration:**

Prospectively registered with the Australian New Zealand Clinical Trial Registry ACTRN12616000304437 on 08 March 2016, https://anzctr.org.au/Trial/Registration/TrialReview.aspx?id=369867.

## Background

Borderline personality disorder (BPD) commonly has its onset during adolescence or early adulthood (young people) [1] and affects an estimated 1–3% of young people in the community, 11–22% of outpatients, and 33–49% of inpatients [2, 3]. BPD is characterised by extreme sensitivity to perceived interpersonal slights, an unstable sense of self, intense and volatile emotions, and impulsive behaviour [4]. BPD is associated with severe and enduring functional disability [5], physical ill-health [6], co-occurring mental-state disorders [7], and high direct healthcare resource use and costs [8]. Structured psychological interventions have consistently demonstrated clinically significant improvement among young people with BPD [9], yet the evidence for pharmacotherapy remains inconclusive [10].

Given the nature of the disorder and its association with various adverse long-term outcomes for individuals, it is unsurprising that relatives, partners, and friends (carers) of adults with BPD report considerable adversity. Qualitative research highlights the experience of chronic and traumatic stress, burden, prolonged hopelessness, a stigmatising healthcare system, shrinking social networks, and feelings of grief, guilt, and distress experienced by carers of adults with BPD [11–14]. Quantitative studies demonstrate that carers of adults with BPD experience higher rates of psychological symptoms and distress than the general population [15]. Burden among carers of adults with BPD has been reported to be even greater than that associated with other severe mental disorders [16, 17]. This also includes elevated objective and subjective burden, grief, impaired ‘empowerment’ (e.g., difficulties interacting with the mental health service system), and mental health problems, including depression and anxiety [16]. Parents of daughters diagnosed with BPD reported experiencing significant burden in multiple domains that include emotional and physical health problems and marital difficulties [18].

Carers of adults with BPD also report family environments high in expressed emotion, particularly criticism and emotional overinvolvement [19]. Past research has found that family environments with high expressed emotion are deleterious to the clinical outcome of patients with psychotic [20], depressive [21], bipolar [22], and eating disorders [23]. Research among patients with BPD has demonstrated that criticism and hostility do not predict clinical outcomes and that emotional overinvolvement predicts better clinical outcomes [24]. This has led to speculation that emotional overinvolvement might be experienced as validating for patients with BPD and therefore supportive of recovery. Importantly, high expressed emotion is correlated with increased burden and mental health problems among carers of adults with BPD [19], suggesting that expressed emotion has differential effects for patients and their carers.

There is limited research examining the experiences of carers of young people with BPD features. A study of 23 carers of young people with BPD features [25] found that carers reported ‘moderate’ levels of psychological distress and elevated burden, comparable with that experienced by carers of adults with BPD [16]. Carers of young people with other mental disorders, including first-episode psychosis (FEP; [26, 27]) and eating disorders [28, 29], and diseases such as cancer [30], also report elevated levels of distress, negative caregiving experiences, maladaptive coping, and/or family environments characterised by criticism and emotional overinvolvement. It is unclear how these experiences compare with those of carers of young people with BPD.

This study aims to: (i) characterise the distress, caregiving experiences, coping, and expressed emotion reported by carers of young people with BPD features; and (ii) compare this with published data from groups of carers of young people with other severe illnesses and with adults from the general population.

## Methods

### Participants

*‘BPD carers’* comprised 82 carers (M age = 44.74, SD = 12.86) of 54 outpatient young people with BPD *‘yp-BPD*’. The yp-BPD were aged between 15 and 25 years (M age = 18.76, SD = 3.02) and fulfilled three or more DSM-IV BPD criteria, as assessed with the Structured Clinical Interview for DSM-IV Axis II Disorders (SCID-II) [31];.

Convenience c*omparison groups,* comprising adults from the general population and carers of young people with other severe illnesses, were drawn from existing literature (Table 1).

**Table 1:** Comparison Group Characteristics and Scores on Measures

Measure	Comparison group	Method of data collection for comparison group	Mean (range) age of young person	Mean (standard deviation) score on measure of comparison group
Kessler Psychological Distress Scale (K-10)	Australian adults (*N* = 8841; [32])	Face-to-face interview	–	14.50 (9.40)
	Carers of young people diagnosed with cancer (*N* = 204; [30])	Questionnaires distributed to and returned by carers via post	21.60 (15–25)	18.05 (7.98)
	Carers of young people with first-episode psychosis (*N* = 124; [26])	Telephone interview	– (15-25)	22.50 (12.25)
Experiences of Caregiving Inventory - Positive Scale (ECI-P)	Carers of young people with anorexia nervosa (*N* = 71; [29])	Self-report questionnaires	24.00 (−)	28.00 (8.80)
	Carers of young people with first-episode psychosis (*N* = 63; [27])	Face-to-face interview	20.11 (15–25)	29.45 (2.45)
Experiences of Caregiving Inventory - Negative Scale (ECI-N)	Carers of young people with anorexia nervosa (*N* = 71; [29])	Self-report questionnaires	24.00 (−)	84.00 (35.00)
	Carers of young people with first-episode psychosis (*N* = 63; [27])	Face-to-face interview	20.11 (15–25)	73.65 (6.65)
Coping Inventory for Stressful Situations - Task-Oriented Scale (CISS-T)	Canadian adults (*N* = 483; [33])	Self-report questionnaires	–	57.87 (11.05)
Coping Inventory for Stressful Situations - Emotion-Oriented Scale (CISS-E)	Canadian adults (*N* = 483; [33])	Self-report questionnaires	–	40.87 (11.82)
Coping Inventory for Stressful Situations - Avoidance-Oriented Scale (CISS-A)	Canadian adults (*N* = 483; [33])	Self-report questionnaires	–	43.96 (11.10)
Family Questionnaire - Critical Comments Scale (FQ-CC)	Carers of young people with a diagnosed eating disorder (*N* = 193; [28])	Self-report questionnaires	21.30 (−)	23.40 (5.50)
	Carers of young people with first-episode psychosis (*N* = 63; [27])	Face-to-face interview	20.11 (15–25)	21.55 (1.30)
Family Questionnaire - Emotional Overinvolvement Scale (FQ-EOI)	Carers of young people with a diagnosed eating disorder (*N* = 193; [28])	Self-report questionnaires	21.30 (−)	28.20 (4.40)
	Carers of young people with first-episode psychosis (*N* = 63; [27])	Face-to-face interview	20.11 (15–25)	25.10 (1.15)

*Note.* “–” = data not reported or not applicable

### Measures

All measures were well-established, reliable and valid self-reports [33–35], rated on a Likert scale. The 10-item *Kessler Psychological Distress Scale* (K-10; [36]) captured distress. The 66-item *Experience of Caregiving Inventory* (ECI; [35]) measured positive (ECI-P) and negative (ECI-N) experiences of caregiving. The 48-item *Coping Inventory for Stressful Situations* (CISS; [33]) assessed task-oriented (CISS-T), emotion-oriented (CISS-E), and avoidance-oriented (CISS-A) coping. Reduced CISS-T and increased CISS-E coping are positively correlated with depression and anxiety whereas increased CISS-A coping is negatively correlated with depression [37]. The 20-item *Family Questionnaire* (FQ; [34]) measured expressed emotion, specifically critical comments (FQ-CC) and emotional overinvolvement (FQ-EOI). All measures demonstrated good to excellent internal consistency in this sample (Cronbach’s alpha 0.80 to 0.96).

### Procedure

Data for both BPD groups were collected at baseline, as part of a randomised controlled trial evaluating psychoeducation interventions for carers of *yp-BPD* [38]. Consecutive sampling was employed to reduce sampling bias [39]. Specifically, at the commencement of recruitment, *BPD carers* of all currently registered *yp-BPD* were invited into the study, then *BPD carers* of all consecutive new referrals were invited to participate. Data for the comparison groups were drawn from representative and relevant comparable published data available for other illness groups, where similar measures had been applied. Independent-samples t-tests were conducted using IBM® SPSS® Statistics Version 22. Cohen’s *d* was calculated with effect sizes defined as ‘small’ (0.2); ‘medium’ (0.5); and ‘large’ (0.8) [40].

## Results

### Demographic, diagnostic and caregiving experience characteristics

*BPD carers* ranged in age from 15 to 74 years, most were female and the mother of the *yp-BPD*, had secondary or lower education, were employed, and were in a relationship (Table 2).

**Table 2:** Demographic Characteristics for Carers of Young People with BPD features (BPD carers)

Characteristic	Descriptive Statistic	Total Sample *N* = 82
Age in years	*M* (*SD*)	44.74 (12.86)
Female	% (*n*)	70.73 (58)
Relationship to young person	% (*n*)	
Mother		51.22 (42)
Father		17.07 (14)
Sibling		8.54 (7)
Partner		6.10 (5)
Other		17.07 (14)
Education	% (*n*)	
Secondary or Lower		48.78 (40)
University		39.02 (32)
Other		12.20 (10)
Employment	% (*n*)	
Employed		62.20 (51)
Unemployed		15.86 (13)
Homemaker/Carer		10.97 (9)
Other		10.97 (9)
Marital status	% (*n*)	
In a relationship		57.32 (47)
Not in a relationship		42.68 (35)

*Note. M* = mean; *SD* = standard deviation

Tables 3 and 4 comprise the demographic and diagnostic characteristics of the *yp-BPD* and the descriptive statistics of the *BPD carers* on each measure, respectively.

**Table 3:** Demographic and Diagnostic Characteristics of the Young People with BPD Features (yp-BPD)

Characteristic		Total Sample (*N* = 54)
Age in years	*M* (SD)	18.76 (3.02)
Female	% (*n*)	75.93 (41)
Young people with *≥*5 DSM-IV BPD criteria	% (*n*)	47.92 (23)
SCID-II PQ BPD total score^a^	*M* (SD)	11.96 (2.15)
SCID-II BPD criteria^b^	*M* (SD)	4.67 (1.21)
Number of concurrent mental state disorders	*M* (SD)	1.74 (0.87)

*Note.*^a^Scores ranged from 6 to 15; ^b^Scores ranged from 3 to 8; *M* = mean; SD = standard deviation; DSM-IV = Diagnostic and Statistical Manual of Mental Disorders 4th Edition; BPD = borderline personality disorder; SCID-II = Structured Clinical Interview for DSM-IV Axis II Disorders; PQ = Personality Questionnaire

**Table 4:** Means and Standard Deviations for Caregiving-Experience Variables (N = 82)

Measure	Mean (SD)
Psychological Distress (K-10)	24.99 (8.83)
Negative Experiences of Caregiving (ECI-N)	104.92 (33.86)
Positive Experiences of Caregiving (ECI-P)	26.98 (7.96)
Task-oriented Coping (CISS-T)	49.87 (10.54)
Emotion-oriented Coping (CISS-E)	42.40 (12.57)
Avoidance-oriented Coping (CISS-A)	38.85 (9.26)
Critical Comments (FQ-CC)	24.70 (6.46)
Emotional Overinvolvement (FQ-EOI)	27.78 (5.80)

### Psychological distress

The mean K-10 score was over one standard deviation higher than that of general population adults [32], which was significant with a very large effect size, *t* (8921) = 10.07, *p* < .001, *d* = 1.15. The mean K-10 score was also significantly higher than that of carers of young people with cancer [30], *t* (281) = 6.45, *p* < .001, *d* = 0.83, but not significantly different to that found among carers of young people with FEP [26], *t* (204) = 1.60, *p* = .112, *d* = 0.23.

### Experiences of caregiving

The mean ECI-N score was significantly higher than that of carers of young adults with anorexia nervosa [29], *t* (151) = 3.75, *p* < .001, *d* = 0.61; and carers of young people with FEP [27], *t* (143) = 7.21, *p* < .001, *d* = 1.28. The mean ECI-P score was not significantly different to that of carers of young adults with anorexia nervosa [29], *t* (151) = 0.74, *p* = .463, *d* = 0.12; or of carers of young people with FEP [27], *t* (143) = 1.41, *p* = .159, *d* = 0.25.

### Coping

Compared with general population adults [33], the mean CISS-T score in this sample was significantly lower, *t* (563) = 6.01, *p* < .001, *d* = 0.73, the mean CISS-E scorewas not significantly different, *t* (563) = 1.07, *p* = .284, *d* = 0.13, and the mean CISS-A score was significantly lower, *t* (563) = 3.90, *p* < .001, *d* = 0.49.

### Expressed emotion

The mean FQ-CC score was not significantly different to that of carers of young adults with an eating disorder [28], *t* (273) = 1.70, *p* = .091, *d* = 0.22; but was significantly higher than that of carers of young people with FEP [27], *t* (143) = 2.25, *p* = .026, *d* = 0.37. The mean FQ-EOI score was not significantly different to that of carers of young adults with an eating disorder [28], *t* (273) = 0.62, *p* = .533, *d* = 0.08; but was significantly higher than that of carers of young people with FEP [27], *t* (143) = 2.17, *p* = .032, *d* = 0.35.

## Discussion

This study has demonstrated that carers of young people with BPD features experience elevated levels of distress, negative experiences of caregiving, and expressed emotion and tend to engage in maladaptive coping, compared with carers of young people with other severe illnesses or with general population adults. Specifically, carers of young people with BPD reported significantly higher levels of psychological distress compared with carers of young people with cancer and general population adults, greater negative experiences of caregiving compared with carers of young people with anorexia nervosa and FEP, reduced task- and avoidance-oriented coping compared with general population adults, and higher levels of expressed emotion compared with carers of young people with FEP.

The high levels of psychological distress reported in the current study are consistent with previous research on carers of young people [25] and adults [15–17, 19] with BPD. However, direct comparisons are limited due to the different measures of psychological distress utilised across studies. Based on normative data [41], carers in the current study reported average K-10 scores indicative of a 58.9% probability of meeting criteria for a current DSM-IV mental disorder and a 69.4% probability of meeting criteria for a DSM-IV mental disorder sometime in the previous 12 months. This is consistent with previous research demonstrating that 64.2% of carers of adults with BPD endorsed symptoms consistent with threshold mood or anxiety disorders [19]. That a majority of carers of both young people and adults with BPD report high levels of distress indicative of an underlying mental disorder suggests that carers might benefit from treatment interventions in addition to psychoeducation and practical support. Further research might also explore whether there are reliable differences in carer distress across the course of BPD, which might inform more targeted interventions at different stages in the trajectory of the disorder.

In this study, carers reported significantly greater negative, but comparable positive, experiences of caregiving, compared with carers of young people with anorexia nervosa or FEP. Further research is needed to clarify the specific factors contributing to this finding. Possible variables might include the extent to which these disorders differ along an externalizing-internalizing continuum, the extent to which carers experience differing levels of distress and mental health problems, differences in the explanatory models utilised by carers to understand these disorders, and the extent to which the disorders are differentially stigmatized among healthcare professionals and the broader community. For instance, Hoffman et al. [42] report that carers of individuals with BPD experience a sense of “surplus stigma” or stigma that is over and above what is typically experienced by carers of persons with other serious mental illnesses. Examples of this include carers of individuals with BPD being told by some mental health professionals, “I don’t accept BPDs in my practice,” or “We don’t want those patients in our hospital” [42] (page 223). Surplus stigma might be one factor exacerbating negative caregiving experiences for carers of young people with BPD.

Of interest is that carers in the current study reported comparable positive caregiving experiences to those caring for young people with anorexia nervosa or FEP. Research into the positive aspects of caregiving has been largely neglected and future studies should aim to capture a broader range of caregiving experiences including positive experiences that might also be responsive to intervention and lead to better outcomes for carers and patients.

Compared with general population adults [33], carers in this study reported reduced task-oriented coping, which has been demonstrated to be positively correlated with depression and anxiety, and reduced avoidance-coping, which has been positively correlated with depression [37]. Carers reported normative levels of emotion-oriented coping. These findings suggest that carers of young people with BPD features utilise coping strategies associated with increased psychopathology and might benefit from training in more adaptive coping techniques. Indeed, Pearce et al. (2017) provided preliminary evidence for the benefit of psychoeducation and skills training for carers of young people with BPD features [25]. They found that carers reported reduced subjective, but not objective, burden and increased personality disorder knowledge at the completion of a three session cognitive analytic therapy-informed program called ‘Making Sense of BPD’. The findings have led to a randomised controlled trial evaluation of the program [38]. Future studies are needed to examine the effects of increasing adaptive coping skills on carer and patient outcomes.

Carers in the current study reported high levels of criticism and emotional overinvolvement [34] and this is consistent with previous research examining expressed emotion in carers of adults with BPD [19]. Specifically, carers in the current study reported higher levels of expressed emotion compared with carers of young people with FEP but not compared with carers of young adults with eating disorders. It is unclear what variables might be underpinning these differences and further research examining the role of expressed emotion in carer and patient outcomes is indicated. Existing research has found that family environments characterised by high levels of expressed emotion predict poorer clinical outcomes for patients with mental state disorders [20–23]. For patients with BPD, criticism has been shown to be unrelated to clinical outcome whereas greater emotional overinvolvement predicts better outcomes [24]. It has been speculated that high emotional overinvolvement might be experienced as validating by patients with BPD therefore contributing to their recovery [24]. However, high levels of criticism and emotional overinvolvement predict increased burden and poor mental health among carers of adults with BPD [19]. As such, interventions designed to modify characteristics of the family environment ought to consider possible effects on outcomes for both patients and carers to avoid unwanted harmful effects for either group.

### Strengths and limitations

This study is the first study to compare caring for young people with BPD features with caring for young people in other illness groups. Strengths include the relatively large sample size that is representative of care-seeking young people with BPD features and the use of multiple, gold-standard instruments to capture caregiving experiences and to assess BPD pathology. Limitations include the use of convenience comparison groups, potentially confounding the results due to cohort effects, differences in the operationalisation of ‘carer’, and methods of measure administration. Direct comparisons with carers of adults with BPD would be of interest, but was not possible, as different measures have been employed in research among adults. The cross-sectional study design precludes conclusions regarding causality or potential mechanisms, but this warrants further research. While the 3:1 female to male gender ratio of *yp-BPD* reported in the current study is consistent with previous research in clinical samples [43], population data suggest that there are no significant gender differences [44]. Therefore, it is unclear how these findings might generalise to caring for young males with BPD.

## Conclusions

Carers of young people with BPD features experience considerable adversity, greater than that reported by adults in the general population and similar to or greater than that reported by carers of young people with other severe illnesses. Further research is needed to enhance understanding of the factors underlying this adversity and to develop, evaluate, and disseminate interventions designed to support carers of young people with BPD features. This will require consideration of the needs of carers in research, clinical practice, and policy-making settings.

## Abbreviations

BPD: Borderline personality disorder
DSM-IV: Diagnostic and statistical manual-4th edition
FEP: First-episode psychosis
SCID-II BPD: BPD items from the structured clinical interview for DSM-IV axis II disorders
SCID-II PQ BPD: BPD items from the structured clinical interview for DSM-IV axis II disorders personality questionnaire
K-10: Kessler psychological distress scale
ECI-N: Experience of caregiving inventory - negative subscale
ECI-P: Experience of caregiving inventory - positive subscale
CISS-T: Coping inventory for stressful situations - task subscale
CISS-E: Coping inventory for stressful situations - emotion subscale
CISS-A: Coping inventory for stressful situations - avoidance subscale
FQ-CC: Family questionnaire - critical comments subscale
FQ-EOI: Family questionnaire - emotional overinvolvement subscale
HYPE: Helping Young People Early
yp-BPD: Young people with BPD features
